# Expression of Macrophage Scavenger Receptor (MSR1) in Peripheral Blood Cells from Patients with Different Respiratory Diseases: Beyond Monocytes

**DOI:** 10.3390/jcm11051439

**Published:** 2022-03-05

**Authors:** Selene Baos, Lucía Cremades-Jimeno, María López-Ramos, María Ángeles de Pedro, Silvia A. Uriarte, Joaquín Sastre, Nicolás González-Mangado, María Jesús Rodríguez-Nieto, Germán Peces-Barba, Blanca Cárdaba

**Affiliations:** 1 Immunology Department, IIS-Fundación Jiménez Díaz-UAM, 28040 Madrid, Spain; selenebmuniz@gmail.com (S.B.); lucia.cremades@quironsalud.es (L.C.-J.); maria.lramos@quironsalud.es (M.L.-R.); mpedrm1@gmail.com (M.Á.d.P.); 2 Allergy Department, University Hospital Fundación Jiménez Díaz, 28040 Madrid, Spain; silvia_uriarte@hotmail.com (S.A.U.); jsastre@fjd.es (J.S.); 3 Ciber de Enfermedades Respiratorias (CIBERES), 28029 Madrid, Spain; ngonzalez@fjd.es (N.G.-M.); mjrodriguezn@fjd.es (M.J.R.-N.); gpeces@fjd.es (G.P.-B.); 4 Pulmonology Department, University Hospital Fundación Jiménez Díaz, 28040 Madrid, Spain

**Keywords:** asthma, biomarker, COPD, cytometry, gene expression, macrophage scavenger receptor, MSR1

## Abstract

Background: Macrophage scavenger receptor 1 (MSR1) has mostly been described in macrophages, but we previously found a significant gene expression increase in peripheral blood mononuclear cells (PBMCs) of asthmatic patients. Objective: To confirm those results and to define its cellular origin in PBMCs. Methods: Four groups of subjects were studied: healthy controls (C), nonallergic asthmatic (NA), allergic asthmatic (AA), and chronic obstructive pulmonary disease (COPD) patients. RNA was extracted from PBMCs. MSR1 gene expression was analyzed by RT-qPCR. The presence of MSR1 on the cellular surface of PBMC cellular subtypes was analyzed by confocal microscopy and flow cytometry. Results: MSR1 gene expression was significantly increased in the three clinical conditions compared to the healthy control group, with substantial variations according to disease type and severity. MSR1 expression on T cells (CD4^+^ and CD8^+^), B cells, and monocytes was confirmed by confocal microscopy and flow cytometry. In all clinical groups, the four immune cell subtypes studied expressed MSR1, with a greater expression on B lymphocytes and monocytes, exhibiting differences according to disease and severity. Conclusions: This is the first description of MSR1’s presence on lymphocytes’ surfaces and reinforces the potential role of MSR1 as a player in asthma and COPD.

## 1. Introduction

Macrophage scavenger receptor 1 (*MSR1*), also known as SR-A or cluster of differentiation 204 (CD204), is a gene that encodes the class A macrophage scavenger receptors. There are three isoforms: SR-AI and SR-AII are homotrimeric transmembrane proteins, generated by alternative splicing. SR-AIII is translated but not integrated into the membrane. Scavenger receptors were discovered when studying how macrophages take up cholesterol from low-density lipoprotein (LDL) in atherosclerotic plaques from patients with familial hypercholesterolemia [1]. Though MSR1 was considered a receptor specific to macrophages in their first descriptions [2,3,4], more recent publications have described its presence in other types of cells (especially tissue cells) such as vascular smooth muscle cells, endothelial cells, human lung epithelial cells, microglia, astrocytes, and murine embryonic fibroblasts. Few studies using peripheral blood mononuclear cell (PBMC) samples have reported the *MSR1* gene [5,6] or protein [7,8] expression.

MSR1 is implicated in many physiological and pathological processes associated with macrophages, including atherosclerosis and other diseases of the heart, endotoxemia, sepsis, viral infections, host defenses, Alzheimer’s disease, lung injury, and bone metabolism [9]. MSR1 is considered a subclass of pattern-recognition receptors (PRRs) [10,11], an indication of its importance in innate immunity. The MSR1 ligand repertoire includes modified self molecules (considered danger-associated molecular patterns or DAMPs) and non-self molecules or pathogen-associated patterns (PAMPs). Additionally, MSR1 has the ability to partner with co-receptors, which endows them with versatility in functional responsiveness in homeostasis and also enables them to fight infection. This versatility can explain the controversial association between scavenger receptors and macrophage polarization, defined mostly in M2 macrophages (anti-inflammatory effects), although, in certain contexts, they can also appear in M1 macrophages (pro-inflammatory responses). Thus, MSR1 can join other receptors, forming complexes, and induce inflammatory or anti-inflammatory responses depending on the context [12]. It was demonstrated that MSR1 can associate with MER receptor tyrosine kinase (MERTK) in murine macrophages and form a complex that promotes the clearance of apoptotic cells [13]. However, MSR1 can also partner with the Toll-like receptor 4 (TLR4) in macrophages in the presence of bacterial lipopolysaccharides (LPS), thus activating the nuclear factor-κB (NF-κB) pathway [14]. This has created controversy concerning the dual role played by MSR1 in different diseases [15,16,17,18,19,20,21,22,23,24,25,26,27]. The mechanisms underlying this variable behavior remain unclear. Kelley et al. [9] concluded that depending on the type of activator ligand, MSR1 might trigger the shift from cell survival to death, highlighting the conflicting role that MSR1 plays in health and disease.

With regard to the respiratory system, several studies have found an association between polymorphisms [28,29] and overexpression [30] of *MSR1* in human macrophages from chronic obstructive pulmonary disease (COPD). In hyperoxia-induced and ovalbumin-induced asthma in murine models, the presence of SR-A in lung tissue was found to exert a protective effect [31,32]. In this respect, our group demonstrated the relevance of gene and protein MSR1 expression in asthmatic disease in PBMC samples. *MSR1* was the most overexpressed gene in severe nonallergic asthma (NA) compared to healthy controls; its presence at the protein level was confirmed, and interesting differences between NA and allergic asthmatic (AA) subjects were discovered [33,34,35,36].

Given the scarcity of studies about MSR1 expression in PBMCs and its potential association with different pathologies, including respiratory diseases, the present study aimed to corroborate the expression of MSR1 in PBMCs, determine its association with asthma and COPD, and characterize the PBMCs’ cellular subpopulations that express this receptor.

## 2. Materials and Methods

### 2.1. Study Design: Subjects

The study population comprised 46 unrelated subjects: 11 healthy control (C) subjects, 11 patients with nonallergic asthma (NA), 13 patients with allergic asthma (AA) (allergic to airborne allergens), and 11 patients with chronic obstructive pulmonary disease (COPD).

Inclusion criteria were:

Male or female aged 18–70 years;

Asthma or EPOC confirmed diagnosis by allergist or pneumologist.

The control group has to be healthy subjects with no history of respiratory diseases or allergic symptoms.

Willingness and ability to provide written informed consent.

Exclusion criteria were:

Do not have the right range of age;

Current malignancy or immunological treatments.

The subjects were diagnosed in the Allergy and Pulmonology Departments of the Fundación Jiménez Díaz (FJD) Hospital (Madrid, Spain), and all samples were processed in the Immunology Department of the Health Research Institute-FJD-UAM (IIS-FJD-UAM). Patients were diagnosed as having severe, moderate, or mild asthma according to the Spanish Guidelines for the Management of Asthma (GEMA) [37], and COPD severity was classified according to the GOLD (Global Strategy for the Diagnosis, Management, and Prevention of COPD) classification system [38]. Tests of pulmonary function were carried out by determining the predicted percentage of forced vital capacity (% FVC) and the forced expiratory volume in 1s (% FEV1). All the Cs and patients were tested by skin prick tests against a panel of standardized common allergens (ALK-Abelló, Madrid, Spain), including mites (*Dermatophagoides pteronyssinus*, *Dermatophagoides farinae*, and *Lepidoglyphus destructor*), epithelia (cat and dog), cockroaches (*Blatella orientalis* and *Blatella germanica*), pollens (*Cypress*, *banana shadow*, olive, a mixture of grasses, *Artemisia*, *Parietaria*, and *Salsola*), and fungi (*Alternaria*, *Cladosporium*, *Aspergillus*, and *Penicillium*). The C group was composed of healthy subjects with no history of respiratory diseases or allergic symptoms. The NA patients were diagnosed with asthma disease but had a negative skin prick test, while the AA patients were asthmatic and allergic to at least one airborne allergen. The COPD subjects did not present asthma or allergic diseases. Written informed consent was obtained from each subject in accordance with the Declaration of Helsinki, and ethical approval for the study was obtained from the research ethics committee of the IIS-FJD-UAM.

### 2.2. Isolation of PBMCs and RNA Extraction

PBMCs were isolated from heparin-containing peripheral blood samples by gradient centrifugation using Lymphoprep (Comercial Rafer, Zaragoza, Spain) following the manufacturer’s instructions. Cells isolation was performed under sterile conditions using endotoxin-free reagents. RNA was isolated from 1 to 5 × 10^6^ PBMCs using the TRIzol reagent (Invitrogen, Carlsbad, CA, USA). RNA quantification and purity was checked using a spectrophotometer (Nanodrop ND-1000, Bonsái Technologies Group, Madrid, Spain).

### 2.3. Differential Gene Expression Analysis

Gene expression analyses were performed by quantitative real-time PCR (RT-qPCR). Briefly, reverse transcription of 800 ng of RNA from each subject was performed using the High-Capacity RNA-to-cDNA kit (Applied Biosystems, Foster City, CA, USA). Then, qPCR was performed using the TaqMan Gene Expression kit and the 7500 Real-Time PCR System (Applied Biosystems) with 40 amplification cycles, to study in triplicate the gene expression of *MSR1* and *18S* as the reference gene, using the predesigned TaqMan Gene expression assays *MSR1*-Hs00234007_m1 and *18S*-Hs99999901_s1, respectively. Relative Quantification (RQ) values were calculated according to the cycle threshold (Ct) method, with the gene expression represented as 2^−ΔΔCt^, where ΔΔCt = (ΔCt (clinical group)) − (ΔCt (control group)) and ΔCt = (Ct (MSR1)) − (Ct (18S)).

### 2.4. MSR1 Expression in Peripheral Samples by Confocal Microscopy and Flow Cytometry

MSR1 expression in different cell populations of PBMCs was analyzed by confocal microscopy and by flow cytometry. For both techniques, 100 µL (10^6^ cells) of PBMCs in BSA 1%-PBS1x were first incubated with the primary antibody for MSR1 (rabbit anti-human polyclonal IgG CD204 antibody (PA522956), 1:100 dilution), manufactured by Thermo Fisher Scientific, Rockford, IL, USA, and then with a goat anti-rabbit IgG secondary antibody tagged with FITC (dilution, 1.5:100) (Santa Cruz Biotechnology, Dallas, TX, USA). A control of the secondary antibody was performed, by incubating the cells only with the secondary antibody. Additionally, simultaneous staining was performed for four cell populations with their corresponding antibodies (BD Bioscience, Franklin Lakes, NK, USA): a mouse anti-human monoclonal IgG1, κ antibody specific for human CD4 labeled with PE (dilution, 1:50) to detect T CD4^+^ lymphocytes; a mouse anti-human monoclonal IgG1 antibody specific for human CD8 labeled with PE (dilution, 1:10) for T CD8^+^ lymphocytes; a mouse anti-human monoclonal IgG1, κ antibody specific for human CD19 labeled with PE (dilution, 1:10) for B lymphocytes; and a mouse anti-human monoclonal IgG2 antibody specific for human CD14 labeled with PC7 (dilution, 1:20) to characterize monocytes. Specific isotype controls from BD were used according to the manufacturer’s protocols (BD Bioscience) for each fluorescent antibody. For confocal microscopy, 400 µL of resuspended cells labeled with the specific antibodies in PBS1x were visualized in suspension in an 8-well microscope plate (Ibidi, Martinsried, Germany) using a Leica SP5 confocal microscope (Leica Microsystems, Wetzlar, Germany). Images were taken with an oil immersion objective (40×) using the LAS AF Leica Microsystems program. Fluorescence measurement for cytometry analysis was performed with cells labeled with the specific antibodies and resuspended in 100 µL of PBS1x and 200 µL of FACS Flow (Fluorescence-Activated Cell Sorting Flow) buffer (BD Bioscience) and captured with a FACS Canto II flow cytometer (BD Bioscience). The results, expressed as the percentage of cells expressing MSR1 normalized to the total percentage of cells within each subpopulation (i.e., MSR1^+^cells in CD4^+^ T-Lymphocytes: % cells double MSR1^+^CD4^+^/% total CD4^+^ cells), were determined using the BD FACS Diva program (BD Bioscience).

### 2.5. Statistical Analysis

Differences in gene expression, using the ΔCt values, and the percentages of cells expressing MSR1 among groups were compared, using GraphPad InStat 3. First, a one-way analysis of variance (ANOVA) was carried out and, when this test showed statistically significant differences, two-by-two comparisons were performed with the unpaired *t*-test. Statistical significance was established as a two-tailed *p* value < 0.05. Additionally, a stricter threshold was established for gene expression at an RQ < −2 or >2.

## 3. Results

### 3.1. Subjects

The demographic and clinical characteristics of the study population are summarized in Table 1. There were statistically significant differences in age between the patients with COPD and with asthma, with the COPD patients being the oldest and the AA patients being the youngest. In all groups, more than 80% were women, except the COPD subjects, all of whom were men. Future research including female COPD patients and a more balanced age population should be performed in order to analyze the possible confounding aspects of gender and sex.

Nearly all subjects were non-smokers or ex-smokers. Patients were selected according to disease severity: half had a severe diagnosis (of asthma or COPD), and the other half were moderate-to-mild cases. Of the patients with NA, 36.36% also presented nasal polyposis, 27.27% nonallergic rhinitis, 9.09% sinusitis, 9.09% heart disease, and 9.09% hypothyroidism. The concomitant diseases of AA patients were: 30.77% rhinitis; 15.39% esophageal reflux; 7.69% nasal polyposis; 7.69% eosinophilic esophagitis; 7.69% thyroid goiter; and 7.69% ulcerative colitis. Among the COPD patients, 9.90% presented rhinitis. The two groups with asthma had a significantly higher percentage of forced expiratory volume in 1s (FEV_1_) when compared to the COPD group. In terms of severity, there were statistically significant differences in lung function in the AA group (%FEV_1_ in severe: 60.03 ± 19.70% vs. moderate–mild: 94.32 ± 13.73%, *p* = 0.0044; predicted percentage of forced vital capacity (%FVC) in severe: 73.39 ± 18.43% vs. moderate–mild: 112.32 ± 9.51, *p* = 0.0007). In the COPD subjects, relevant differences were also found in the percentage of FEV_1_ (severe: 34.75 ± 11.41% vs. moderate–mild: 65.80 ± 18.54%, *p* = 0.0225). No statistically significant differences were observed in the patients with NA, but the moderate–mild NA patients tended to have higher values for lung function parameters (%FEV_1_ severe NA: 75.50 ± 24.58%, moderate-mild NA: 94.07 ± 20.65%; %FVC severe NA: 93.25 ± 15.84%, moderate-mild NA: 101.28 ± 13.90%).

### 3.2. Gene expression Analysis

**Figure 1:**
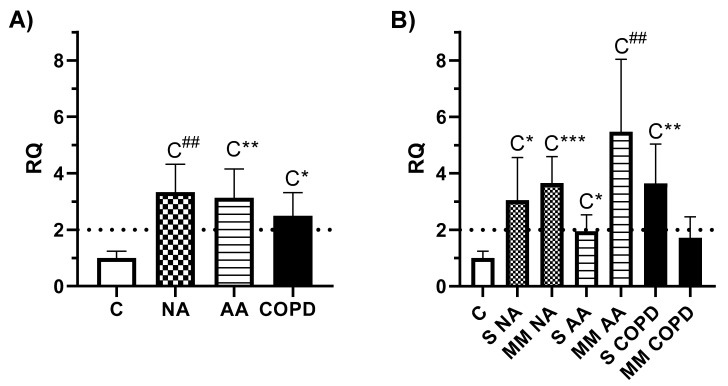
*MSR1* gene expression according to clinical group, and severity of disease. C: Control (*n* = 11), NA: Nonallergic Asthma (*n* = 11), AA: Allergic Asthma (*n* = 13), COPD (*n* = 11), S: severe diagnosis; MM: moderate–mild diagnosis. (**A**) Gene expression results according to clinical group. The graphs represent the gene expression of *MSR1*, with relative quantification (RQ) for the clinical groups compared to the C group. Error bars represent the standard error. The dotted line indicates an RQ value equal to 2. Statistically significant differences between C and the indicated disease group are shown as: C* (*p* < 0.05), C** (*p* < 0.005) and C^##^ (*p* < 0.001). (**B**) Gene expression results according to the severity of disease. Statistically significant differences between C and the indicated disease group are shown as: C* (*p* < 0.05), C** (*p* < 0.005), C^##^ (*p* < 0.001) and C*** (*p* = 0.0005).

MSR1 gene expression was analyzed in all subjects of the study. First, the ANOVA test showed variation among at least two of the clinical groups (*p* = 0.0067) and severity groups (*p* = 0.0034); thus, a deeper study was performed. The clinical groups showed a gene overexpression of *MSR1* compared to the C group (Figure 1A): NA vs. C (RQ = 3.34, *p* = 0.0007), AA vs. C (RQ = 3.13, *p* = 0.0036), and COPD vs. C (RQ = 2.50, *p* = 0.0120), but without statistically significant differences between groups of disease. The gene expression analysis according to severity (Figure 1B) showed that all the patient groups had higher *MSR1* gene expression than the C. Severe NA (RQ = 3.05, *p* = 0.0162), moderate–mild NA (RQ = 3.65, *p* = 0.0005), moderate–mild AA (RQ = 5.47, *p* = 0.0008), and severe COPD (RQ = 3.64, *p* = 0.0022) followed our statistical criteria (RQ > 2, *p* < 0.05) and the difference between the C and severe AA groups was statistically significant (*p* = 0.0378), but with a lower RQ value (RQ = 1.94).

### 3.3. Expression of MSR1 on Different Subpopulations of PBMCs

The presence of MSR1 on peripheral cells was analyzed by confocal microscopy and flow cytometry to both characterize its origin in distinct cellular populations in PBMC samples and to determine differences according to clinical phenotypes.

#### 3.3.1. MSR1 Expression in PBMCs by Confocal Microscopy

**Figure 2:**
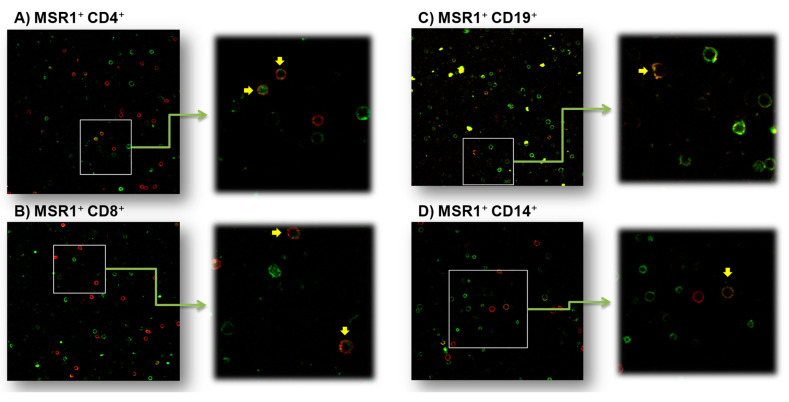
Representative example of PBMCs marked with fluorescence for the subpopulations: (**A**) CD4^+^, (**B**) CD8^+^, (**C**) CD19^+^, and (**D**) CD14^+^ and the MSR1 protein. Confocal microscopy visualization (*n* = 3). Images on the left were obtained with a 40× objective, and those on the right, with 2× zoom. MSR1^+^ cells are labeled in green, each labeled cellular subpopulation is indicated in red, and the colocalization of the corresponding cellular subpopulation and MSR1^+^ is marked in yellow. The yellow arrows indicate cells with double labeling.

MSR1 was expressed on cell membranes of the four subpopulations of PBMCs analyzed (CD4^+^ and CD8^+^ T lymphocytes, B lymphocytes (CD19^+^ cells), and monocytes (CD14^+^ cells)) as shown by the colocalization of the MSR1 and the specific CD markers. Figure 2 shows a representative example of the images obtained, in which the combined labeling of the CD of each population in red and of MSR1 in green is verified in some of the cells. These results corroborate the presence of MSR1 in the membranes of the PBMC subpopulations studied.

#### 3.3.2. MSR1 Expression in PBMCs by Flow Cytometry

**Figure 3:**
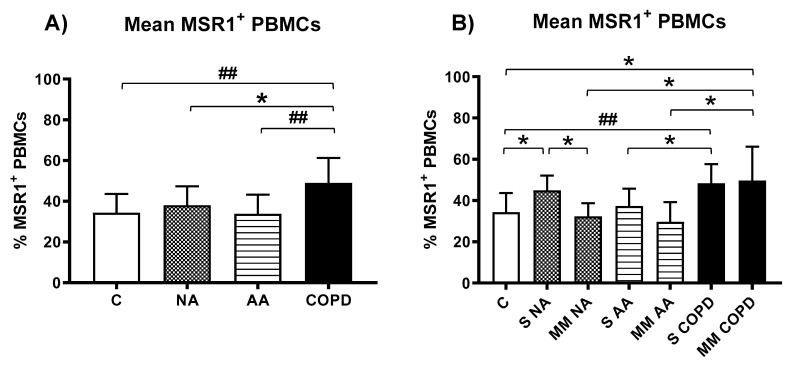
Expression of MSR1 on PBMCs. C: Control (*n* = 11), NA: Nonallergic Asthma (*n* = 11), AA: Allergic Asthma (*n* = 13), COPD (*n* = 11), S: severe diagnosis; MM: moderate–mild diagnosis. Mean percentages of total MSR1^+^ expressing cells in the whole PBMCs fraction (**A**) by clinical group and (**B**) according to disease severity. Statistically significant differences among groups are shown as: * (*p* < 0.05) and ## (*p* < 0.001).

The MSR1 expression in the total PBMCs and in four cellular subpopulations was determined by flow cytometry. A representative example of isotype controls and cytometry results is described in the Supplementary Results (Figure S1). First, the percentages of positive cells for MSR1 in the total fraction of PBMCs were studied for each of the four clinical groups (Figure 3A) and according to disease severity (Figure 3B), showing statistically significant differences in both cases.

The mean total percentages of MSR1^+^ positive cells were higher in the COPD group than the others (COPD group: 48.97 ± 12.31% vs. C group: 34.36 ± 9.30%, *p* = 0.0051, vs. NA group: 38.05 ± 9.22%, *p* = 0.0288, and vs. AA group: 33.81 ± 9.42%, *p* = 0.0025) (Figure 3A).

According to the severity of asthma and COPD, the severe NA group (44.98 ± 7.14%) had higher amounts of MSR1^+^ cells than the C (34.36 ± 9.30%, *p* = 0.0408) and moderate–mild NA groups (32.27 ± 6.42%, *p*= 0.0125). This expression was also significantly higher in the severe COPD (48.37 ± 9.27%, *p* = 0.0095) and moderate–mild COPD groups (49.70 ± 16.44%, *p* = 0.0302) than the C group (Figure 3B). Statistically significant differences were also found between distinct phenotypes and severities, such as the COPD group with moderate–mild diagnoses vs. the patients with moderate–mild NA (*p* = 0.0394) and moderate–mild AA (29.62 ± 9.57%, *p* = 0.0319), and severe COPD vs. severe AA (37.40 ± 8.27%, *p* = 0.0454) (Figure 3B).

**Figure 4:**
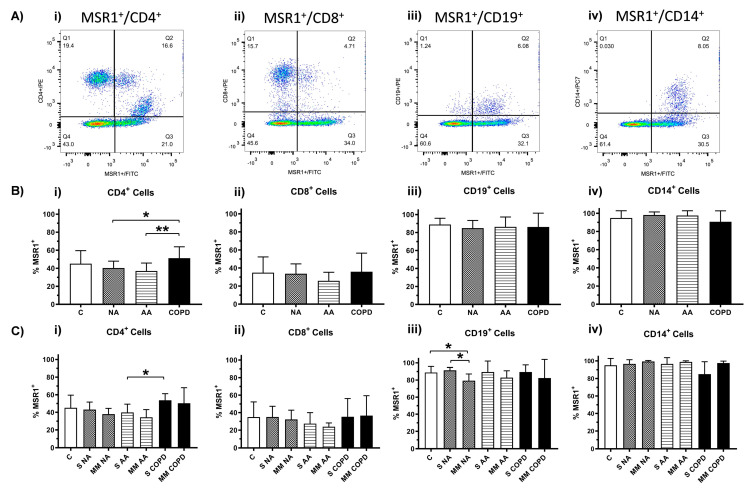
Expression of MSR1 on cell subpopulations of PBMCs. C: Control (*n* = 11), NA: Nonallergic Asthma (*n* = 11), AA: Allergic Asthma (*n* = 13), COPD (*n* = 11), S: severe diagnosis; MM: moderate–mild diagnosis. (**A**) Representative example of the results obtained in cell subpopulation PBMCs analysis: (**i**) MSR1^+^ CD4^+^ double positive cells; (**ii**) MSR1^+^ CD8^+^ double positive cells; (**iii**) MSR1^+^ CD4^+^ double positive cells; and (**iv**) MSR1^+^ CD4^+^ double positive cells. (**B**) Expression of MSR1^+^ cells by clinical groups. Mean percentages of MSR1^+^ cells within (**i**) CD4^+^ subpopulation, (**ii**) CD8^+^ subpopulation, (**iii**) CD19 subpopulation, and (**iv**) CD14^+^ subpopulation. (**C**) Mean percentages of total MSR1^+^ cells according to disease severity. Mean percentages of MSR1^+^ cells within (**i**) CD4^+^ subpopulation, (**ii**) CD8^+^ subpopulation, (**iii**) CD19 subpopulation, and (**iv**) CD14^+^ subpopulation. *,** Statistically significant differences (*p* < 0.05, *p* < 0.005, and *p* < 0.001, respectively) between the indicated groups.

Next, to determine the specific immune cells that express MSR1, PBMCs were analyzed in terms of their expression on four cellular subpopulations: CD4^+^ and CD8^+^ T lymphocytes, B lymphocytes (CD19^+^ cells), and monocytes (CD14^+^ cells). A representative example of double positive (specific CD and MSR1^+^) is shown in Figure 4A. The distribution of the cell subpopulations in isolated PBMC samples by groups is described in the Supplementary Results (Table S1). The analysis of CD4^+^ T cells showed that there were statistically significant differences in the percentages of MSR1^+^CD4^+^ cells when the clinical groups were compared. The COPD group showed a higher percentage of expression (51.17 ± 12.70%) than the groups with asthma (NA group: 40.27 ± 7.64%, *p* = 0.0262; AA group: 37.03 ± 8.77%, *p* = 0.0047) (Figure 4B(i)). In the other cellular subtypes (Figure 4B(ii)–B(iv)), no differences were found between diseases, even though the higher percentages of MSR1 positive cells were found in B-lymphocytes (CD19^+^) and monocytes (CD14^+^). Interestingly, we observed a remarkable percentage of cells expressing MSR1 that did not correspond to any of the cellular subpopulations analyzed in this study, especially in the NA and COPD groups, but we only found significant differences (*p* = 0.0268) between the NA (12.64 ± 16.32%) and AA (1.57± 3.96%) groups (Figure S2A).

According to severity, significant differences were found for MSR1 expression by CD4^+^ T lymphocytes (Figure 4C(i)) and B lymphocytes (Figure 4C(iii)). MSR1^+^CD4^+^ cells were higher in patients with severe COPD (53.70 ± 7.50%) than the severe AA group (39.87 ± 9.43%, *p* = 0.0185). On the other hand, MSR1^+^CD19^+^ cells were found to be lower in patients with moderate–mild NA (79.32 ± 7.78%) than in the C group (88.88 ± 6.95%) and NA patients with severe diagnoses (91.40 ± 3.25%) (*p* = 0.0200 and 0.0105, respectively). Again, we found a small percentage of cells expressing MSR1 that did not correspond to any of the cellular populations analyzed in this study, especially in severe COPD and in both NA severities, but with no significant differences among groups (Figure S2B).

## 4. Discussion

The heterogeneity of asthma has caused management of the disease to be insufficient in certain patients, especially those with severe diagnoses. As such, the identification of reliable biomarkers is essential for the development of treatments based on precision medicine [39,40,41,42].

The aim of this study was to validate previous results from our group relating MSR1 with asthma and, due to its description mainly as a receptor expressed on macrophages or tissue cells, to characterize their cellular origin in peripheral samples. *MSR1* was one of the most overexpressed genes in the PBMCs of a population of AA subjects [43], despite the fact that it had never been associated with asthma. Later, the expression of this gene was described as one of the most differential in asthma, and was mainly associated with the severity of patients with NA [33]. Surprisingly, several studies have suggested *MSR1* as a candidate gene for another complex respiratory disease, COPD [28,29], and previous research has pointed out that the mechanisms that cause accumulation in the lung of MSR1-expressing macrophages could contribute to severe emphysema and COPD [30]. Given our findings regarding its expression in asthma and in light of data from COPD patients, the design of this study included samples of subjects that presented NA, AA, and COPD. Gene analyses of MSR1 were carried out to gather information on its expression in PBMCs. Again, the two groups with asthma showed higher *MSR1* gene expression than healthy controls (Figure 1A) in all the clinical phenotypes, although this seemed to be higher in the group with moderate–mild AA diagnosis. Interestingly, the COPD patients also showed a significant increase compared to the C group, with higher *MSR1* expression in the severe patients (Figure 1A). Differences between clinical groups evidenced only tendencies, without statistically significance, most likely due to the small size of patients in the severity-based group for each disease.

*MSR1* is a hypoxia-induced gene reported to be involved in the clearance of pathogens and apoptotic cells. In another respiratory disease, acute respiratory distress syndrome (ARDS) caused by SARS-CoV infection, whose course is also characterized by lung fibrosis, *MSR1* overexpression in lung-tissue samples was associated with late-stage disease, with more severity and where lung fibrosis was consolidated [44]. This increase was interpreted as playing a role in adapting the cells to oxidative stress and a hypoxic environment, a common occurrence in SARS-CoV-infected patients.

Few published studies have analyzed the expression of MSR1 on the surface of PBMCs, and those that have evidenced expression have done so in subsets of monocytes in diseases such as acute coronary syndromes [6] and in systemic sclerosis [7]. Moreover, a novel subpopulation of PBMCs was found that expresses MSR1 and markers of fibrocytes and M2 macrophages, which seem to be the origin of dermal fibrosis-creating scars in patients presenting severe burns [8]. This study tries to define subpopulations of PBMCs that could be responsible for the *MSR1* gene expression differences detected in asthma and COPD. For this purpose, MSR1 expression was analyzed in the total PBMC fraction, as well as in the four main cellular subpopulations in PBMCs: CD4^+^ and CD8^+^ T lymphocytes, B lymphocytes, and monocytes. First, the MSR1 expression on the surface of cellular subpopulations was demonstrated by confocal microscopy (Figure 2). Next, to quantify the MSR1 expression, flow cytometry was used. The percentages of cells expressing MSR1 in the total PBMC fraction were considerable in all clinical study groups (Figure 3), with several statistically significant differences between groups. Patients with COPD had the highest percentage of MSR1-positive cells (Figure 3A), thus confirming in peripheral samples what was described in tissue samples by other authors [28,29,30]. Further characterizing disease severity, the results in Figure 3B seem to corroborate the importance of this biomarker in patients with COPD, though this role also resembles that of MSR1 in NA disease and severity.

Regarding the cell subpopulations, all the cell types studied expressed MSR1, with B lymphocytes (CD19^+^) and monocytes (CD14^+^) displaying the highest percentage (Figure 4). Statistically significant differences between clinical phenotypes were observed in CD4^+^ T lymphocytes (Figure 4B(i),C(i)) and B lymphocytes (Figure 4C(iii)). Differences observed in the percentages of CD4^+^ T lymphocytes according to MSR1 expression were relevant in the comparison of the clinical groups, while differences in B lymphocytes seemed to be associated with severity subgroups. Notably, we found two separated MSR1^+^CD4^+^ cell subpopulations (Figure 4A(i)), which could indicate that there is a second subpopulation of CD4^+^ cells expressing MSR1 in PBMCs, beyond T-lymphocytes. This subpopulation, with a lower CD4 and higher MSR1 expression, probably corresponds to monocytes, a cell subtype in which MSR1 expression has been previously described [7,8], a fact that could overestimate the percentage of MSR1^+^CD4^+^ T cells. Further studies of MSR1 expression in isolated T-lymphocytes would enrich these results.

Despite this limitation and the derived mainly from the size of the sample, we have demonstrated that MSR1 is expressed by four main subpopulations of PBMCs, with several differences between clinical groups. However, in AA patients, the percentage of MSR1 expression in the four cellular subpopulations studied was similar to the total MSR1 expression on total PBMCs (Figure S3), meaning that MSR1 expression is justified by the cells studied. Meanwhile, in patients with NA and COPD, there was a percentage of MSR1^+^ cells that could correspond to other cell types not analyzed here. The expression of MSR1 has been described in fibrocytes [45], controversial cells located in peripheral blood samples; the definition of these cells has been challenging to characterize, and it has recently been associated with uncontrolled asthma, asthma exacerbations, and chronic obstructive asthma [46,47,48]. Furthermore, differences in the number of circulating fibrocytes among patients with severe asthma and patients presenting nonsevere asthma have been demonstrated [49]. Therefore, it would be interesting to demonstrate the expression of MSR1 in the fibrocytes of patients with NA using peripheral blood samples.

Overall, these results provide new evidence of the potential role of MSR1 in patients with asthma and COPD. Additionally, their expression on PBMC subpopulations opens up new study fields of the functional implications of MSR1 in cells other than macrophages and their correlation with disease. The high presence of MSR1 on monocytes, as macrophage precursors, was unsurprising, though the presence of MSR1 on B and T cells, as far as we know, has never been described before. Having in mind the relevance of these receptors in many pathological conditions and their functional versatility, this aspect could be very relevant and should be researched deeper to explain its function in these cells.

## 5. Conclusions

In summary, this study presents promising results, though these should be tested in larger populations. Here, we describe the presence of MSR1 (macrophage scavenger receptor 1), classically associated with macrophage and tissue cells, in several cell populations of PBMCs, specifically in T and B lymphocytes, as well as monocytes. MSR1 gene and protein expression differed according to asthma and COPD clinical phenotype, reinforcing the possible role of MSR1 in these diseases.

## Figures and Tables

**Table 1:** Demographic and clinical characteristics of the study population.

	N	Gender		Age	Smoking Habit			Clinical Diagnosis		%FVC	%FEV_1_
		F (%)	M (%)		NS (%)	S (%)	ES (%)	Asthma/COPD	Allergy		
**Control (C) subjects**	11	81.8	18.2	49.1 ± 14.5 *	91	9.0	0	100% healthy		-	-
**Nonallergic asthmatic (NA) subjects**	11	81.8	18.2	56.2 ± 16.8 *	63.6	9.1	27.3	45.5% SA	No-allergic symptoms	98.1 ± 14.4	86.6 ± 23 *
								54.5% MMA	Negative-skin prick test		
**Asthmatic allergic (AA) subjects**	13	92.3	7.7	41.2 ± 13.5 ^#,†^	76.9	15.4	7.7	53.8% SA	92.3%pollen allergy	91.3 ± 24.8	75.85 ± 24.27 *
								46.2% MMA	7.7% no pollen allergy		
**COPD subjects**	11	0	100	70.4 ± 7.9	9.1	9.1	81.8	54.5% SC	9.1% pollen allergy	76.2 ± 31.8	52 ± 22.1
								45.5% MMC	90.9% no allergies		

F: females. M: males. NS: non-smokers. S: smokers. ES: ex-smokers. SA: severe asthma. MMA: moderate-mild asthma. SC: severe COPD. MMC: moderate-mild COPD. Pollen allergy: allergic to pollen. No pollen allergy: nonallergic to pollen but allergic to other airborne allergens. No allergies: nonallergic to airborne allergens. %FVC: percentage of forced vital capacity; %FEV_1_: percentage of forced expiratory volume in 1 s. * Statistically significant comparison (*p* < 0.05) between the COPD group and the indicated group. ^†^ Statistically significant comparison (*p* < 0.05) between the NA group and the indicated group. ^#^ Statistically significant comparison (*p* < 0.0001) between the COPD group and the indicated group.

## Data Availability

Not applicable.

## Supplementary Materials

The following supporting information can be downloaded at: https://www.mdpi.com/article/10.3390/jcm11051439/s1, Figure S1. Representative example of flow cytometry results of an AA patient in isolated PBMCs; Figure S2: Expression of MSR1 on PBMCs. Table S1: Mean values of the cell subpopulations studied in PBMCs.
