# Supplementary material for: Expression of Macrophage Scavenger Receptor (MSR1) in Peripheral Blood Cells from Patients with Different Respiratory Diseases: Beyond Monocytes
Source: J Clin Med. 2022 Mar 5;11(5):1439. doi: 10.3390/jcm11051439 (PMC8910889; doi:10.3390/jcm11051439)
Supplement: Supplementary file 1 [file jcm-11-01439-s001.zip › jcm-1605457-supplementary.pdf]

Supplementary Material

Results

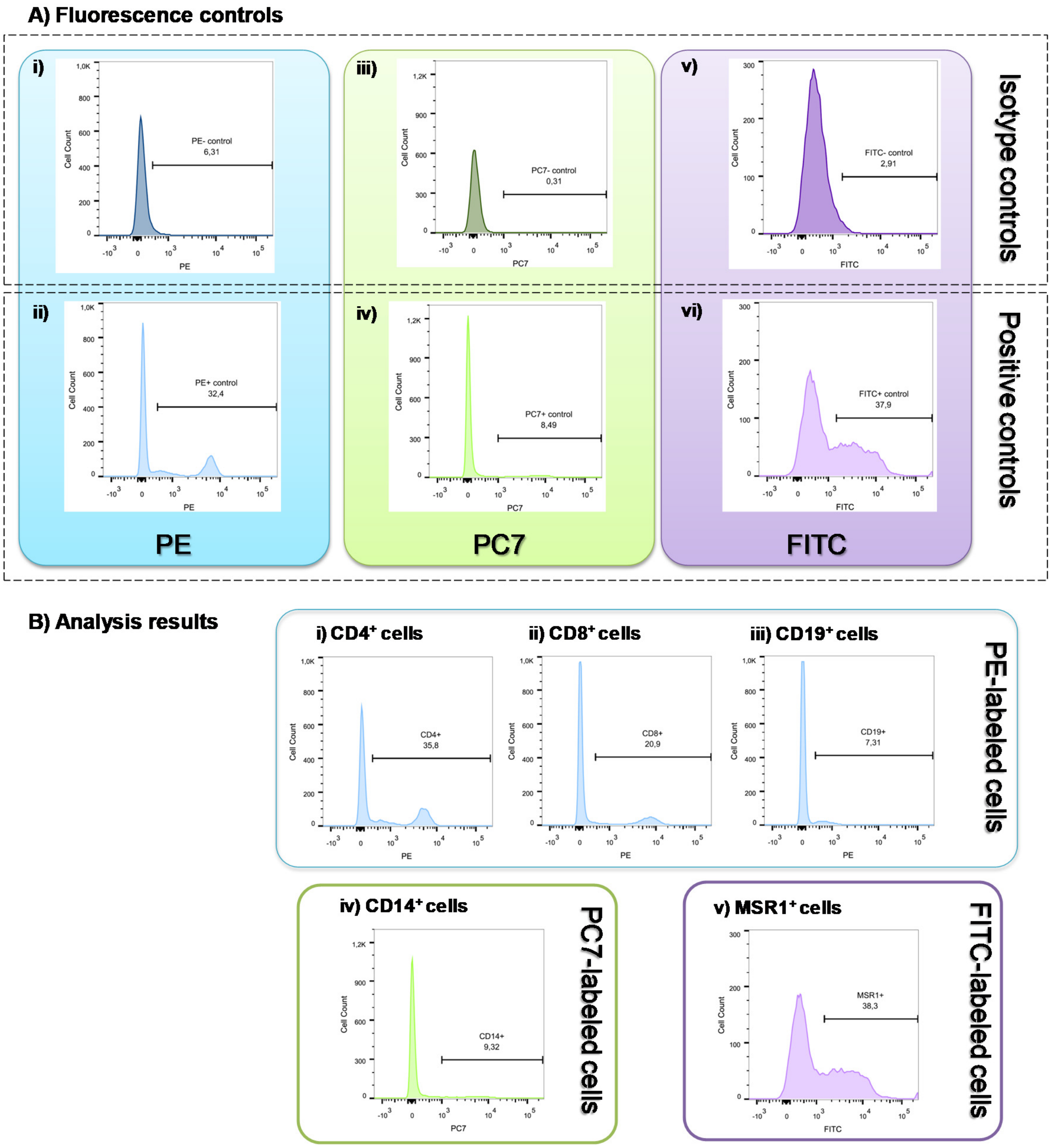

Figure S1. Representative example of flow cytometry results of an AA patient in isolated PBMCs. A) Fluorescence controls. Positive and negative (isotype) controls of the different fluorochromes used: i) PE- and ii) PE+, used for CD4, CD8 and CD19 label; iii) PC7- and iv) PC7+, for CD14 label; and v) FITC- and vi) FITC+, used for the MSR1 label. B) Histogram representation of the results obtained in an AA patient, representing the total cell counts for each subpopulation studied in PBMCs sample: i) $CD4^+$ cells, ii) $CD8^+$ cells, iii) $CD19^+$ cells; iv) $CD14^+$ cells; and v) $MSR1^+$ cells.

Distribution of cell subpopulations in isolated PBMC samples determined by flow cytometry

Tables S1 shows the mean percentages of each cellular subpopulation analyzed ($CD4^+$ and $CD8^+$ T lymphocytes, B lymphocytes, and monocytes) in each clinical phenotype regardless of their MSR1 expression. Statistically significant differences were observed in $CD4^+$ and $CD14^+$ cells when were analyzed according to the severity of the diseases. On the one hand, patients with moderate-mild COPD showed a higher percentage of $CD4^+$ T lymphocytes when compared to severe COPD (p=0.0435), to moderate-mild NA (p=0.0183), and to moderate-mild AA patients (p=0.0023). On the other hand, the percentage of $CD14^+$ cells was significantly higher in the moderate-mild AA group than in the moderate-mild NA group (p=0.0378).

Table S1: Mean values of the cell subpopulations studied in PBMCs

	Severity of disease	%$CD4^+$ cells	%$CD8^+$ cells	%$CD19^+$ cells	%$CD14^+$ cells
**C group (n=11)**		45.94 ± 14.67	20.48 ± 4.26	4.45 ± 1.85	8.12 ± 5.84
**NA group (n=11)**		43.80 ± 12.22	21.59 ± 7.38	5.59 ± 4.24	9.21 ± 4.16
	MM NA (n=6)	38.50 ± 12.25*	19.58 ± 7.59	5.03 ± 2.58	7.90 ± 2.47*
	S NA (n=5)	50.16 ± 9.64	24.00 ± 7.12	6.26 ± 5.98	10.78 ± 5.47
**AA group (n=13)**		43.91 ± 6.99	18.82 ± 6.24	5.17 ± 3.28	12.02 ± 4.24
	MM AA (n=6)	40.92 ± 5.59**	21.42 ± 5.87	4.27 ± 2.93	10.65 ± 1.35
	S AA (n=7)	45.61 ± 8.02	16.59 ± 6.04	5.94 ± 3.59	13.20 ± 5.56
**COPD group (n=11)**		46.84 ± 13.31	19.08 ± 6.40	4.31 ± 2.98	12.21 ± 6.27
	MM COPD (n=5)	55.40 ± 4.95	20.18 ± 2.53	4.96 ± 4.28	10.56 ± 2.02
	S COPD (n=6)	39.70 ± 14.15*	18.17 ± 8.63	3.77 ± 1.54	13.58 ± 8.39

C: Control, NA: Nonallergic Asthmatic, AA: Allergic Asthmatic, COPD: chronic obstructive respiratory disease. MM: moderate-mild diagnosis, S: severe diagnosis. %$CD4^+$ cells column: *Statistically significant comparison ($p<0.05$) between the indicated group and the moderate-mild COPD group. **Statistically significant comparison ($p<0.01$) between the indicated group and the moderate-mild COPD group. %$CD14^+$ cells column: *Statistically significant comparison ($p<0.05$) between the indicated group and the moderate-mild AA group.

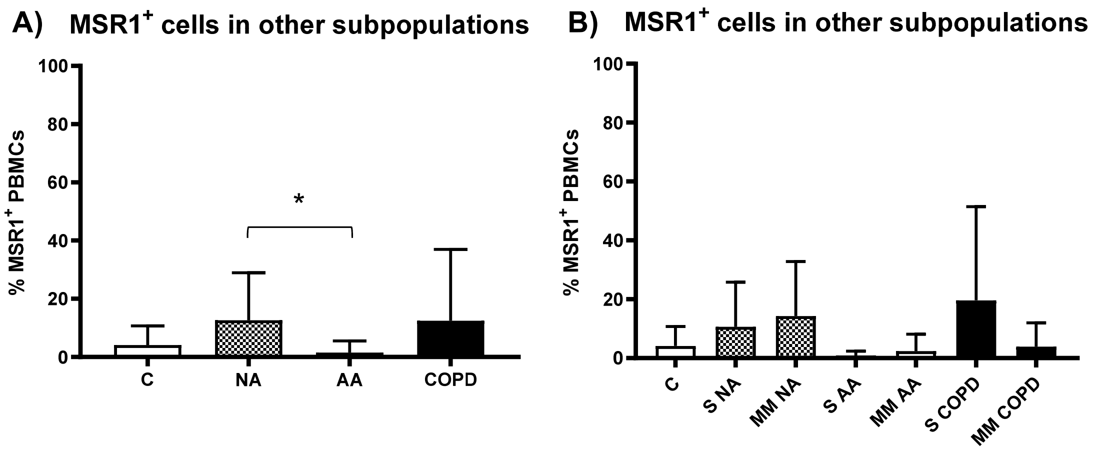

Figure S2. Expression of MSR1 on PBMCs. C: Control (n=11), NA: Nonallergic Asthma (n=11), AA: Allergic Asthma (n=13), COPD (n=11), S: severe diagnosis; MM: moderate–mild diagnosis. Mean percentage of MSR1+ cells within the non-studied cellular subpopulations according to A) clinical groups and B) disease severity. *Statistically significant differences ($p < 0.05$) between the indicated groups.
